# Quantitative histologic evaluation reveals different degree of liver atrophy in cachectic and starved dogs

**DOI:** 10.1177/10406387221128326

**Published:** 2022-10-01

**Authors:** Emanuele Ricci, Ilaria D’Aquino, Orlando Paciello, Vanessa Whitfield, Lorenzo Ressel

**Affiliations:** Department of Veterinary Anatomy, Physiology and Pathology, Institute of Infection, Veterinary and Ecological Sciences, University of Liverpool, Liverpool, United Kingdom; Department of Veterinary Medicine and Animal Production, University of Naples Federico II, Naples, Italy; Department of Veterinary Medicine and Animal Production, University of Naples Federico II, Naples, Italy; Greater Manchester Hospital, RSPCA, Salford, United Kingdom; Department of Veterinary Anatomy, Physiology and Pathology, Institute of Infection, Veterinary and Ecological Sciences, University of Liverpool, Liverpool, United Kingdom

**Keywords:** atrophy, dogs, cachexia, forensic pathology, liver, starvation

## Abstract

Cases of neglect in dogs are among the forensic cases submitted most commonly for postmortem examination. Starvation is a form of primary protein-energy malnutrition in which the availability of food is severely restricted or absent; cachexia is a form of protein-energy malnutrition secondary to progressive metabolic derangement during chronic diseases. Despite both conditions leading to an emaciated appearance of the cadaver, discrimination between the two is crucial in forensic cases. We hypothesized that among emaciated dogs, the degree of liver atrophy in starved animals is higher than in cachectic ones, and that this can be investigated microscopically, regardless of the degree of cadaver decomposition. We studied 46 animals: 23 starved, 11 cachectic, and 12 control dogs. Portal tracts were identified by the presence of a bile duct and associated vascular structures recognizable by a thin rim of collagen still visible regardless of the degree of cadaver decomposition. The number of portal tracts per lpf (10×) was used as an indirect measure of atrophy. The number of portal tracts in starved dogs was significantly higher (*p* < 0.01) compared to both cachectic and control dogs, indicating a higher degree of liver atrophy in starvation. Measuring the density of portal tracts offers a reliable additional tool for discrimination between starvation and cachexia.

Neglect in dogs is among the most common forensic etiologies in cases submitted for postmortem examination.^6,11^ Of >1,200 legal cases submitted to the Department of Veterinary Anatomy, Physiology and Pathology, Institute of Infection, Veterinary and Ecological Sciences, University of Liverpool (DVAPP-UL; Liverpool, United Kingdom) since ~2010, 62% of forensic cases were confirmed as cases of neglect. Among fatal cases of neglect, starvation is a commonly alleged cause of death when an emaciated animal is found dead or agonal (and subsequently euthanized) in suspicious circumstances. It is also important to note that emaciation of the body is the common end-point of multiple conditions, including the outcome of spontaneous diseases.^6^

*Starvation* is a term used to describe the serious and fatal consequences of primary protein-energy malnutrition in which an otherwise healthy animal is able or willing to eat but unable to do so because of the restricted availability of food. Differently from wildlife, for which availability of food is susceptible to seasonal fluctuation,^10^ domestic animals such as dogs rely completely on human caregivers or owners for nutritional support, especially if living in enclosed premises. In contrast, *cachexia* is a term used to describe protein-energy malnutrition consequent to endogenous factors, most significantly represented by chronic inflammation and neoplasia. In such circumstances, the generalized effects of persistently secreted pro-inflammatory cytokines, such as tumor necrosis factor–α and interleukin–1, are the key mediators in the widespread metabolic disarray characterizing cachexia. Cancer cachexia describes the negative cytokine-mediated effect of tumor growth on body mass.^5,9^ Here, we use the term cachexia to define all types of concurrent chronic pathologic processes, such as infections, severe organ degenerations, and neoplasia, which hamper assimilation and utilization of nutrients and are responsible for the shift to a long-term catabolic response.

Starvation and cachexia normally both lead to emaciation; however, their pathogenesis and consequent liver metabolic derangement differ. In starvation, the appetite is preserved, as well as the ability to masticate, swallow, and digest food. Reduction in liver size has been observed in animal models of fasting in which atrophy was found to be the result of a reduction in cell size, noted as soon as 4 d after caloric restriction.^14^ Such a phenomenon is likely the result of the generalized “energy saving” mechanism that affects glandular and epithelium-rich compartments,^6^ prevalently and more promptly. On the contrary, liver atrophy is not reported in humans as being a primary change in cancer cachexia, but liver mass has been observed to paradoxically increase in some cancer cachexia patients.^13^ Liver is a metabolically active organ in cancer cachexia, as also demonstrated in a rat model,^3^ in which mitochondria were described as nutrient-demanding compared to controls, given the pronounced oxidative activity of these cells. It is, however, plausible that in the last stages of cachexia, the organ suffers from generalized negative energy balance, possibly undergoing terminal and genuine atrophy.

Despite both starvation and cachexia causing similar degrees of loss of body mass in emaciated cadavers, starvation is characterized by a prominent decrease of basal metabolic rate with sequential mobilization of lipids and proteins from endogenous sources, and dismantling of skeletal muscles as a source of proteins after exhaustion of fat deposits.^6,10^ In contrast, in cachexia, the animal has a voluntary reduction in caloric intake and/or an increase in metabolic demand with consequent increase in protein catabolism and a disproportionate loss of lean body mass compared to adipose tissue. Cachexia is often refractory to nutritional support alone, whereas starvation is not.^6^

From a legal perspective, it is of paramount importance that the pathologist describes in detail the condition of the cadaver without omission of body compartments or organs, given that the absence of concurrent diseases in the presence of emaciation in a deceased animal may support the diagnosis of starvation.^6^ In other words, death by starvation is a diagnosis by exclusion, supported by the absence of any other relevant and contributory concurrent disease. In dogs, specific findings, such as detection of abnormal gastrointestinal (GI) content (foreign bodies, non-nutritious ingesta), are indicative of preserved appetite and observed in ~50% of animals dead as a result of starvation.^6^ In addition to muscles and adipose tissue, organs reported to undergo atrophy in dogs in both starvation and cachexia include liver, testis, and skin. However, to our knowledge, no quantitative assessment has been reported in dogs to investigate if a difference exists between starvation and cachexia. The liver undergoes atrophy in golden hamster models of starvation,^14^ although microscopic hepatic changes during cachexia have not been studied extensively,^12^ and results are often conflicting. In cancer cachexia, for example, hepatitis is reported more often than hepatic atrophy,^12^ and increased activity and function of the liver is reported in some cases.^13^

We hypothesized that in emaciated dogs, the degree of liver atrophy in starvation cases is higher than in cachexia cases, and that this can be investigated microscopically, regardless of the degree of body decomposition.

## Materials and methods

Autopsy reports of dogs who underwent postmortem examination (gross and histopathology) from January 2014 to April 2018 (*n* = 378) were retrieved from the archives of the DVAPP-UL. Inclusion criteria were as follows:

Control dog group (non-emaciated dogs; WSAVA body score ≥3 of 9)^7^: evaluated clinically if the animal was found alive and euthanized on humane grounds; normal aspect of the cadaver (adequate muscle bulk and presence of adipose tissue); clinical and pathology confirmation of acute deaths.

Cachectic dog group (WSAVA body score <3 of 9)^7^: evaluated clinically if the animal was found alive and euthanized on humane grounds; generalized emaciated aspect of the cadaver (evident bony prominences, in particular scapular spine, ribs, and iliac crest; muscle atrophy and reduction or loss of adipose tissue); a final postmortem diagnosis documenting a neoplastic, chronic inflammatory, or degenerative condition.

Starved dog group (WSAVA body score <3 of 9)^7^: evaluated clinically if the animal was found alive and euthanized on humane grounds; emaciated aspect of the cadaver with a final postmortem diagnosis of starvation based on absence of any chronic pathologic conditions, with muscle atrophy and reduction of loss of adipose tissue, and additional corroborating evidence (e.g., foreign material within the GI tract).^6^

Based on the expected adult weight for each canine breed, dogs were divided into 3 size groups: small (1–10 kg), medium (11–30 kg), and large (>30 kg); for the one crossbred dog, skeletal and body conformation was used to select the most appropriate size class. Dogs’ ages were estimated based on dentition and the overall appearance of the carcass (whitening of the fur), when such information was not provided or unavailable.

All cases had undergone a postmortem examination, which was defined as gross examination of organs in situ, exenterated, and subsequently opened, followed by histologic investigation of the routinely sampled organs as per the forensic protocol of the DVAPP-UL: pituitary gland, thyroid and parathyroid glands, adrenal glands, skin (flank), muscles (quadriceps and triceps, left and right; diaphragm), brain (frontal cortex, hippocampus, cerebellum, medulla oblongata), peripheral nerves (sciatic and brachial plexus, right and left), heart (right and left ventricles, interventricular septum), spleen, mesenteric lymph nodes, bone marrow, stomach, duodenum, jejunum, ileum, colon, liver, pancreas, mesentery, kidneys, urinary bladder, retrobulbar adipose tissue, ovaries or testis (when present). Tissue samples were collected, fixed in formalin, and processed routinely for histopathology.

Per our routine sampling protocol, a large, 1-cm thick, full-thickness slice of liver was cut perpendicularly to the long axis of the left lateral lobe and once fixed for 48–72 h, a 2 × 1 cm rectangular section from the center of the slice was processed and stained routinely with H&E.

The degree of tissue autolysis varied according to the postmortem interval, which was unknown for most of the bodies, from minimal postmortem artefacts (in reasonably fresh cadavers) to marked autolysis (in cadavers presented after a prolonged postmortem interval).^2,4^ Postmortem alterations of the hepatic microanatomy were scored as follows: 1 = mild = minimal changes, including loss of erythrocyte outline and detachment of vascular endothelium in sinusoids, normal cytoplasm and nuclei of hepatocytes visible, with identifiable portal tracts; 2 = moderate = loss of cohesion between hepatocytes, loss of cytoplasm detail, but with nuclei and cellular outline still visible, and identifiable portal tracts; 3 = severe = loss of nuclear details of hepatocytes with only hepatic cords discernible, but still identifiable portal tracts. In particular, the bile ducts within the portal tracts appeared more resilient that surrounding parenchyma to severe autolysis and served as a landmark for the identification of the portal tract. Cases were excluded from the study if the histology of the liver was considered completely compromised (e.g., organ identification was not possible, or portal tracts not discernible).

For each liver section, quantitative histologic examination was performed blindly and independently by 2 board-certified pathologists (E. Ricci, L. Ressel) on 20 randomly selected, non-overlapping, microscopic fields at low-power magnification (10×, 21-mm diameter field of view, ~346 mm^2^), referred to as low power fields (lpfs), using a brightfield microscope (Eclipse 80i; Nikon). Portal tracts were identified by the presence of a bile duct and associated vascular structures (vein and arteries) encased by supporting connective tissue. Portal tracts were counted manually in each lpf; the results of 20 lpfs were averaged per animal and used as an indirect measure of atrophy, with the rationale that the number of portal tracts increased per surface area further to a reduction in size of the lobule. Congestion, degeneration, and fibrosis were assessed as present or absent. A cumulative average per subject was calculated for statistical purposes. Normality was assessed with a Ryan–Joiner test and correlation analyzed with a Pearson correlation test (*p* ≤ 0.05); comparisons of means between groups (control vs. cachexia vs. starvation) were made through 2-sample *t*-tests for samples with normal distribution (v.19; Minitab).

## Results

We included 46 animals: 23 starved, 11 cachectic, and 12 control dogs (Tables 1–3); 19 were female, 27 were male. There were 9 small, 25 medium, and 12 large breed dogs. The median age of dogs with known age was 5.2 y. Among dogs with known age, control dogs were 1–7-y-old (average 3 y); cachectic dogs were 3–15-y-old (average 11 y), and starved dogs were 1–8-y-old (average 3 y). At gross examination, 6 of 23 (26%) dogs in the starved group had non-edible material within the stomach, frequently associated with superficial erosions and ulcerations of the pyloric mucosa.

**Table 1.** Signalment, cause of death, and decomposition score for dogs included in the control group.

Case	Underlying condition or cause of death	Breed	Weight, kg	Breed group	Sex	Age, y	Decomposition score
1	Euthanasia	German Shepherd	30	Large	CM	4	1
2	Skull fracture	Chihuahua	2.2	Small	M	Adult	2
3	Gunshot wounds	Dogue de Bordeaux	38	Large	M	7	1
4	Thoracic trauma	Chihuahua	2	Small	SF	1	2
5	Strangulation	Staffordshire Bull Terrier	16.2	Medium	M	2	2
6	Pyogranulomatous pneumonitis	Cockapoo	1.4	Medium	F	0.2	2
7	Pyometra	Staffordshire Bull Terrier	14.6	Medium	F	9	2
8	Cardiomyopathy	English Bulldog	33	Medium	M	1	2
9	Cardiovascular failure	Great Dane	82	Large	F	4	3
10	Meningoencephalitis	Labrador Retriever	31	Large	M	1	3
11	Thoracic blunt trauma	Yorkshire Terrier	6.8	Small	M	Adult	3
12	Fatal hemorrhagic diathesis	Border Terrier	NA	Medium	M	0.8	3

CM = castrated male; F = female; M = male; NA = not available; SF = spayed female.

**Table 2.** Signalment, cause of death, and decomposition score for dogs included in the cachectic group.

Case	Underlying condition or cause of death	Breed	Weight, kg	Breed group	Sex	Age, y	Decomposition score
1	Right atrial hemangiosarcoma	American Bulldog	17	Large	M	Adult	1
2	Soft tissue sarcoma	Weimaraner	15	Medium	F	15	1
3	Metastatic carcinoma, vaginal leiomyoma, splenic hemangiosarcoma	Labrador cross	8.3	Medium	F	3	1
4	Splenic hemangiosarcoma	Boxer	17.2	Medium	F	12	1
5	Degenerative disk disease	German Shepherd	19	Large	F	12	1
6	Bladder urothelial carcinoma	Bull Mastiff	20.6	Large	F	10	2
7	Ceruminous gland adenocarcinoma	Dalmatian	21	Medium	F	13	2
8	Metastatic mammary carcinoma, vaginal leiomyoma	Staffordshire Bull Terrier	12	Medium	SF	14	2
9	GIST	Yorkshire Terrier	2.9	Small	F	15	2
10	Chronic bacterial osteomyelitis with sepsis	Labrador	19	Large	M	9	2
11	Hepatoid gland carcinoma	Collie	15	Medium	CM	Adult	3

CM = castrated male; F = female; GIST = gastrointestinal stromal tumor; M = male; SF = spayed female.

**Table 3.** Signalment, cause of death, and decomposition score for dogs included in the starved group.

Case	Underlying condition or cause of death	Breed	Weight, kg	Breed group	Sex	Age, y	Decomposition score
1	Starvation	Puggle	4	Small	SF	1	1
2	Starvation	Staffordshire Bull Terrier	8.5	Medium	M	5	1
3	Starvation	American Bulldog	13	Large	F	3	2
4	Starvation	Poodle	1.4	Small	F	3	3
5	Starvation	Staffordshire Bull Terrier	13	Medium	SF	3	1
6	Starvation	Jack Russel Terrier	3.3	Small	M	Adult	2
7	Starvation	Staffordshire Bull Terrier	10	Medium	M	Adult	2
8	Starvation	Staffordshire Bull Terrier	15	Medium	M	Adult	3
9	Starvation	Rottweiler	19	Large	M	Adult	2
10	Starvation	Yorkshire Terrier	3.8	Small	CM	1	1
11	Starvation	Chihuahua	2	Small	M	1	3
12	Starvation	Staffordshire Bull Terrier	12	Medium	SF	Juvenile	2
13	Euthanasia	Bull Mastiff	12.5	Large	CM	1	2
14	Starvation	Staffordshire Bull Terrier	8	Medium	F	Adult	1
15	Starvation	Bull Mastiff	26	Large	M	Adult	1
16	Starvation	Bull Terrier	11	Medium	CM	2	2
17	Starvation	Staffordshire Bull Terrier	9.2	Medium	CM	2	2
18	Euthanasia	Staffordshire Bull Terrier	7.6	Medium	M	5	1
19	Starvation	Staffordshire Bull Terrier	9.2	Medium	M	2	1
20	Starvation	Staffordshire Bull Terrier	8.2	Medium	M	2	2
21	Starvation	English Bull Terrier	12.5	Medium	M	8	3
22	Starvation	English Bull Terrier	16.3	Medium	M	Adult	1
23	Starvation	Springer Spaniel	4	Medium	F	Juvenile	1

CM = castrated male; F = female; M = male; SF = spayed female.

Liver histologic sections were classified according to the degree of postmortem decomposition as follows: 17 cases exhibited mild, 20 moderate, and 9 severe postmortem decomposition (Tables 1–3). Liver congestion was detected in 17 of 46 dogs (6 control, 6 cachectic, and 5 starved dogs). Vacuolar (lipidosis) and/or hydropic degeneration were identified in 3 cachectic dogs and in 2 dogs for both control and starved groups. Mild fibrosis was detected only in one control dog.

**Figure 1.**
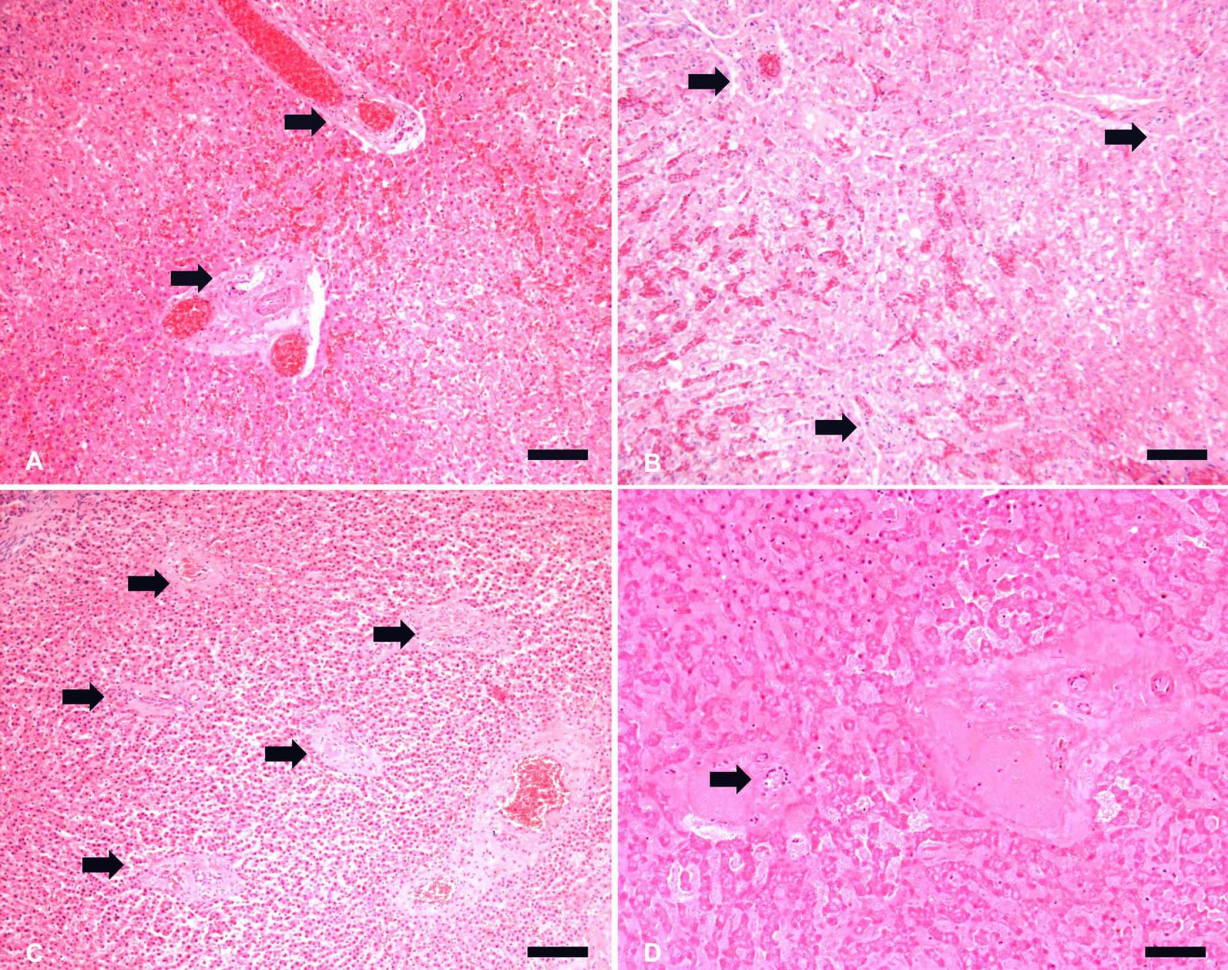
Frequencies of portal tracts among different groups, and postmortem decomposition degrees in canine livers. **A.** Portal tracts (arrows) in histologic section of a dog from the control group. **B.** Portal tracts (arrows) in histologic section of a dog from the cachexia group. **C.** Portal tracts (arrows) in histologic section of a dog from the starved group. **D.** Portal tract (arrow) in histologic section of a dog from the starved group (marked decomposition). Despite postmortem changes, portal tracts are still identifiable. H&E. Bars = 200 μm.

Portal tracts were identifiable in all degrees (mild, moderate, severe) of postmortem decomposition; there was no difference in the average number of portal tracts per field between degrees of autolysis within each experimental group (Fig. 1). We observed no correlation between portal tract numbers and age, sex, body weight, or breed group. No statistical association was observed between the number of portal tracts per field and congestion, degeneration, or fibrosis. Mean values of the number of portal tracts were 3.1 (median 3; SD 0.5) for non-emaciated control cases, 4.1 (median 4; SD 1) for cachectic cases, and 6.9 (median 7; SD 1.6) for starved cases.

**Figure 2.**
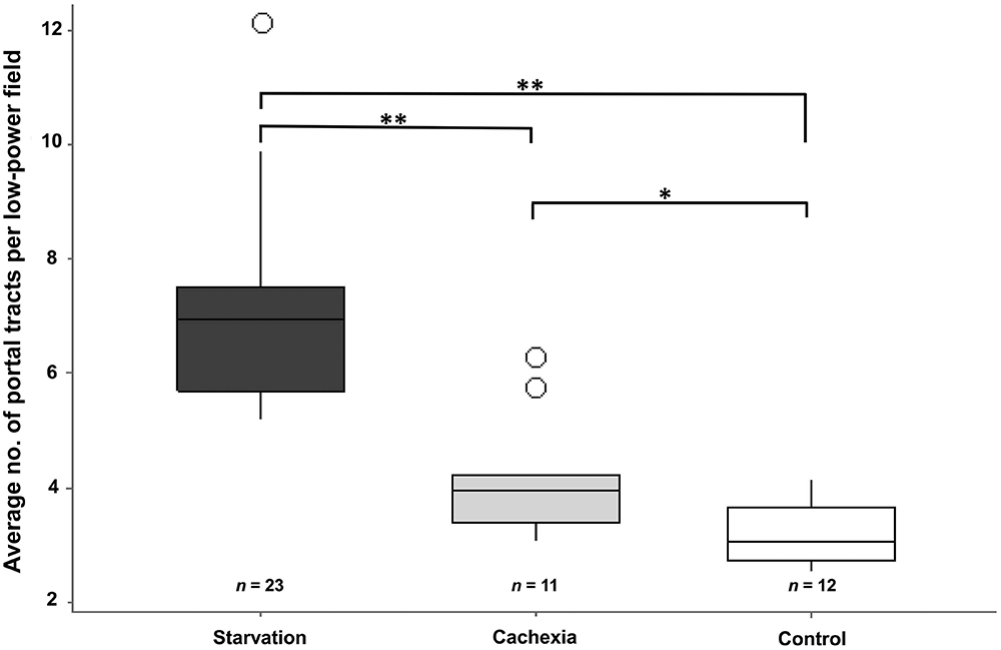
Average numbers of portal tracts per lpf (10×) among groups. There is a highly significant difference (**, *p* < 0.01) between the starved group (*n* = 23) and the cachexia (*n* = 11) and control (*n* = 12) groups, and a significant difference (*, *p* < 0.05) between the cachexia and control groups. Interquartile ranges and medians are represented as box plots and transverse lines, respectively. Vertical lines represent the upper and lower range of data. Circles represent outliers for each group.

The frequency of portal tracts in cachectic (*p* < 0.01) and starved (*p* < 0.01) dogs was significantly higher than control dogs. The frequency of portal tracts was significantly lower in cachectic dogs compared with starved dogs (*p* < 0.01), indicating a higher degree of atrophy in starved compared to cachectic dogs (Fig. 2).

## Discussion

Our comparison by light microscopy of the liver atrophy in cases of starvation and cachexia appears in line with published results,^6^ wherein subjective and qualitative atrophy of the liver was observed in emaciated dogs. In starved dogs, we frequently observed ingested non-edible material within the stomach or intestine, also as reported in the literature.^6^ Ingested material within the stomach, including food as well as non-edible material (plastic, fabric, metal) demonstrates preserved perimortem appetite in the absence of nutritious food. Our first hypothesis was confirmed histologically in our experimental groups; starvation and cachexia resulted in significant hepatic atrophy compared to controls. Indeed, given the use of our quantitative histologic tool, starved dogs had a higher count of portal tracts per lpf compared to cachectic dogs, suggesting that the degree of liver atrophy is objectively higher in starvation compared to cachexia and controls.

Hepatic atrophy during cachexia seems not to reach the degree observed in starvation, at least according to our results. Despite heterogeneity of conditions and tumor types, all diseases affecting the dogs of the cachexia group were presented following a clinical course lasting for weeks and submitted for postmortem examination at the latest stages of their clinical progression. Such a duration in time is deemed to be comparable to the dogs that reached emaciation as a result of starvation. However, despite a similarity in length of clinical course, the reduced degree of hepatic atrophy in cachectic animals indicates that emaciation in cachectic animals is not the simple effect of caloric deficit.

Our histomorphologic study highlighted how, during starvation, the subgross architecture of the liver was maintained, and portal tracts were identified easily. Because of severe atrophy, the diameter of hepatocytes was reduced severely, leading to reduction of the distance between portal tracts, allowing a higher number of visible portal tracts per lpf. We saw no differences in frequency of portal tracts among the 3 categories of postmortem decomposition within each group of dogs. This observation is of pivotal importance considering that most veterinary forensic pathology cases are in various, even advanced, states of decomposition when submitted for examination.^7^

Congestion in histologic sections did not seem to substantially affect the number of portal tracts observable per lpf. We found no significant correlation between lobule size and presence of fibrosis and degeneration. However, the degree of such changes in our population was never marked. We speculate that severe fibrosis, congestion, or hepatocellular degeneration may have an influence on lobular size, therefore careful judgement should be used.

Other quantitative approaches have been adopted to assess liver atrophy, including cell or nuclear size^1^; however, our method is less time consuming and easily reproducible in any standard pathology department. Our approach may be more robust compared to the evaluation of single hepatocyte shrinkage,^8^ which relies on the assessment of entire individual functional units, whose size is affected by both parenchymal and stromal components, rather than by zonal differences in metabolic activity of lobular hepatocytes. A system based on routine H&E and brightfield microscopy is also favored in any standard diagnostic pathology department, allowing a more rapid and consistent approach to quantitative determination of liver atrophy, indirectly determined by the shrinkage in lobule size rather than individual hepatocyte size.

Postmortem changes have been investigated in a rat model, and shrinkage of subcapsular hepatocytes at 72 h was referred to as atrophy.^8^ We believe that, given that this change happened postmortem, atrophy is potentially an incorrect term. However, this “shrinkage” only affected a small layer of the subcapsular hepatic parenchyma, and it would be unlikely to affect the size of the lobules in the interior of the organ, at the site of our evaluation.

A limiting factor of our study is the variability of postmortem decomposition affecting microscopic morphology. This is unfortunately a non-controllable parameter, linked with the wide range of levels of tissue preservation found in forensic cases. Despite this, we demonstrated that identification of portal tracts, using bile ducts as microanatomic landmarks, seems not to be affected by postmortem decomposition. In our sampled population, the degree of postmortem decomposition could be deemed equivalent to grade 3 of the 1–5 scoring system published in human literature.^2^ It is then still plausible that, with higher degrees of postmortem decomposition, the investigator may fail to identify some portal tracts; deploying special stains for collagen could offer valuable assistance in performing such a task. It is important to underline that, when hampered by severe decomposition, the total count of portal tracts would be underestimated, failing to support a potential true starvation case. Therefore, regardless of the state of decomposition, we discourage the over-reliance on this tool alone to issue a diagnosis of starvation, but instead we suggest its use only as an additional and complementary tool during a detailed postmortem examination, as expected in every forensic case, regardless of the specific legal standard of proof.

Furthermore, specific liver conditions, such as primary or secondary hypoperfusion of the portal vein, can produce subtle lesions and mimic the lobular size reduction seen in hepatic atrophy, thus care should be exercised when assessing the degree of liver atrophy using the lpf count to indicate starvation. Careful examination to rule out subtle organ-specific signs (e.g., microvascular dysplasia = increased arteriolar profiles and fading of hepatic veins) and concurrent lesions in other organs are crucial steps in the diagnostic workup, especially so in cases of advanced autolysis.
